# Composing On-Program Triggers and On-Demand Stimuli into Biosensor Drug Carriers in Drug Delivery Systems for Programmable Arthritis Therapy

**DOI:** 10.3390/ph15111330

**Published:** 2022-10-27

**Authors:** Yan Yik Lim, Ahmad Mujahid Ahmad Zaidi, Azizi Miskon

**Affiliations:** 1 Faculty of Defence Science and Technology, National Defence University of Malaysia, Sungai Besi Prime Camp, Kuala Lumpur 57000, Malaysia; 2 Faculty of Engineering, National Defence University of Malaysia, Sungai Besi Prime Camp, Kuala Lumpur 57000, Malaysia

**Keywords:** exogenous triggers, endogenous stimuli, drug delivery systems, biosensors, drug carriers, pathological alterations

## Abstract

Medication in arthritis therapies is complex because the inflammatory progression of rheumatoid arthritis (RA) and osteoarthritis (OA) is intertwined and influenced by one another. To address this problem, drug delivery systems (DDS) are composed of four independent exogenous triggers and four dependent endogenous stimuli that are controlled on program and induced on demand, respectively. However, the relationships between the mechanisms of endogenous stimuli and exogenous triggers with pathological alterations remain unclear, which results in a major obstacle in terms of clinical translation. Thus, the rationale for designing a guidance system for these mechanisms via their key irritant biosensors is in high demand. Many approaches have been applied, although successful clinical translations are still rare. Through this review, the status quo in historical development is highlighted in order to discuss the unsolved clinical difficulties such as infiltration, efficacy, drug clearance, and target localisation. Herein, we summarise and discuss the rational compositions of exogenous triggers and endogenous stimuli for programmable therapy. This advanced active pharmaceutical ingredient (API) implanted dose allows for several releases by remote controls for endogenous stimuli during lesion infections. This solves the multiple implantation and local toxic accumulation problems by using these flexible desired releases at the specified sites for arthritis therapies.

## 1. Introduction

Programmable arthritis therapy [1] is in high demand now because the current therapy still lacks efficacy [2] and no recovery therapy has been discovered. Arthritis therapy is complex, intertwined, and progressively influenced by rheumatoid arthritis (RA) and osteoarthritis (OA) [3]. The most difficult part is the progression of pathological inflammation from OA to RA, which involves mediators, such as synovial hyperplasia and pannus synovitis [4]. Generally, RA and OA are chronic and joint-degenerative diseases, respectively, that are classified as inflammatory arthritis (IA) [5] and non-IA [6], respectively. Commonly, OA and RA are specified for large joints of the hip and knee, and small joints of the hands and wrists, such as metatarsophalangeal and interphalangeal toe joints, respectively [7]. The symptoms of OA begin with joint pain, cartilage degeneration, and osseous overgrowth, whereas RA inherits the OA characteristics and then progresses to cartilage destruction and function loss [7]. These severely restrict the patients’ mobility and activities, resulting in disability and living quality deterioration, respectively.

Although many approaches, such as drug administration routes [8], intra-articular therapies [9], resections [10], reconstruction surgeries [11], and articular cartilage scaffolds [12], have been undertaken, the problems of drug delivery systems (DDS), such as mediate infiltration, pharmacokinetics, pharmacodynamics, efficacy, systemic toxicity, and navigation control, still remain unsatisfactory. Moreover, arthritis therapy involves multiple inflammatory factors, resulting in difficult medication [13]. Therefore, the current therapy is limited to alleviating these disease symptoms [14] or pathological alterations. Nonetheless, they have the same pathological microenvironmental changes that could be used as alterations to design biosensors in DDS [15]. These unique alteration features could be characterised as biosensors [16] in scaffold structures [17], which act as detectors to be sensitively cleaved by the selective endogenous stimuli for active pharmaceutical ingredient (API) releases [18]. As a result, an alternative tissue engineering approach with a better sustained-release feature in DDS holds tremendous promise in target-specified delivery, on-demand stimulation, on-program triggering, and great flexibility [19]. This should be urgently developed towards achieving therapy efficacy and patient compliance in clinical translations [20].

In this review, a comprehensive timeline of DDS is provided to enrich our understanding of the unremitting amelioration discoveries and seminal clinical advancements in the laboratory. Then, the status quo of DDS is summarised with the major clinical triumphant achievements towards their applications. The disputed issue of internal and external controls is reconstructed to elaborate precisely on the mechanisms of on-demand stimuli and on-program triggers, respectively. Of note, the endogenous stimuli are induced by pathological alterations to provide API to the lesion for disease therapy. The exogenous triggers are controlled by external remotes to cleave biosensors for endogenous stimulation. This on-program trigger, like an on-off switch, unlocks the hydrophilic conjugation to enable the endothelial leakiness or endogenous stimulus to release API. This is rational for a guiding therapy to synthesise a drug carrier that is sensitively triggered to induce miscellaneous stimuli to controlled release locally for sustained efficacy and minimal adverse effects. Herein, it is notable that the design of DDS consists of four on-demand stimuli and four on-program trigger mechanisms. However, the relationships between these stimulus mechanisms have remained unclear, which has resulted in a major obstacle towards clinical translation. Through this review, we expect the relationships to be clearly elucidated. That is to say, the key irritant biosensors [21] are acting to stimulate these mechanisms, which are expected to serve as a guiding document aiming to inspire the next-generation DDS innovations.

## 2. Status Quo of Drug Carriers for Arthritis Therapy

The drug carrier for arthritis therapy is progressing from traditional to systemic drug administration routes [8]. The traditional drugs are non-steroidal anti-inflammatory drugs (NSAID) and glucocorticoids, which are commonly used in traditional drug administration routes, such as oral, topical, transdermal, and injection [22]. The systemic drug administration routes, such as intra-articular, surgery, and drug carriers, use API such as disease-modifying anti-rheumatic drugs (DMARD), disease-modifying OA drugs (DMOAD), and biological response regulators [23]. However, the traditional intra-articular therapies, resection, and reconstruction surgeries are still unsatisfactory due to the infiltration, efficacy, drug clearance, and target localisation ability. In particular are the radio-, chemo-, immune-, and hormone therapies after osteosarcoma and tumour surgeries, which may result in systemic side effects [24] such as secondary injury, increased risk of recurrence, and immunological rejection [25,26]. Over a long period of therapy, these may increase the detoxification burdens of the liver, kidney, and bone marrow system, resulting from the toxic doses accumulated [27]. Due to these numerous potential systemic side-effects, it is a complication of therapeutic efficacy that limits their clinical applications. Therefore, a new class of drug carriers with better DDS is continuously being created.

### 2.1. Historical Development

**Figure 1:**
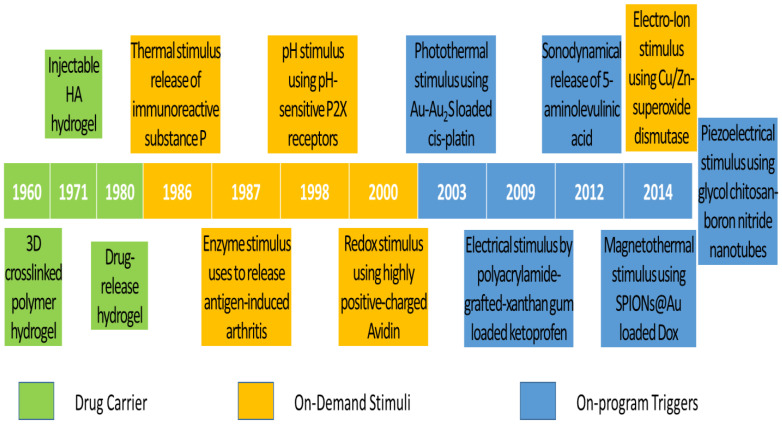
Timeline of significant discoveries and advancements in drug delivery systems with drug carriers, on-demand stimuli, and on-program triggers.

The historical development of DDS is highlighted with a timeline of their significant discoveries and advancements, as shown in Figure 1. The first drug carrier as hydrogel, mostly used in DDS, was invented by Wichterle and Lím in 1960 via a 3D cross-linked polymer, as shown in the green background in Figure 1 [28]. This hydrogel has become one of the most popular drug carriers because of its nanosize particles, API affinity-based nature, bioactive release control, and injectable designs. The first injectable hydrogel using hyaluronic acid (HA) was invented by Rydell and Balazs in 1971 [29]. Later, HA was most commonly used in pain relief for OA therapy. Until 1980, the hydrogel was used as DDS, becoming popular after a drug-release device with rate-controlling was invented by Lee et al. [30]. Even though many thermal therapies were used before 1986, the thermal stimulus was first introduced by Helme et al., which was used to release immune-reactive substance P for inflammation therapies, as shown in the orange background in Figure 1 [31]. In 1987, the enzymatic stimulus using antigen-induced arthritis was first introduced by Bonanomi et al. to investigate the pharmacokinetics and therapeutic effects [32]. In 1998, the pH stimulus using pH-sensitive P2X receptors [33] was first introduced by Dowd et al. to study peripheral nociceptive afferents in the rat knee joint [34]. P2X receptors are the membrane ion channels that open in response to the binding of extracellular adenosine 5′-triphosphate (ATP). In 2000, the redox stimulus using Cu/Zn-superoxide dismutase (SOD) loaded with Fc receptor was first introduced by Vouldoukis et al. to study apoptosis [35]. In 2014, the electro-ion stimulus using electrostatic interaction between negatively charged proteoglycans and highly positively charged Avidin was first introduced by Bajpayee et al. to investigate its pharmacodynamics effects [36].

The existing DDS still does not meet the demands of scientists and researchers. Therefore, more flexible drug release systems or those composed of the existing trigger systems are being developed. Ren and Chow (2003) used a photothermal trigger with Au-Au_2_S-loaded cis-platin for near infra-red (NIR) DDS, as shown in the blue background in Figure 1 [37]. This DDS was a composed stimulation of photo and thermal, where gold linkages were leaked by NIR to free cis-platin for thermal stimulation. In 2009, Kulkarni and Sa used an electrical trigger with polyacrylamide-grafted xanthan-gum-loaded ketoprofen for transdermal DDS [38]. It was a simple study of in vitro drug release influenced by an electrical trigger. In addition, in 2012, Lv et al. used a sonodynamical trigger of 5-aminolevulinic acid therapy to investigate anti-carcinoma efficiency [39]. In these in vivo and in vitro studies using low-intensity US vibration, they found efficacy for SAS cell line proliferation and apoptosis. However, it is regretful that the increment of ROS in the therapy process was not further investigated. In 2014, Mohammad and Yusof developed a magnetothermal trigger using gold-coated superparamagnetic iron oxide nanoparticles (SPIONs@Au) loaded with doxorubicin (Dox) as an anthracycline-type chemotherapy drug for cancer therapy [40]. This in vitro DDS found that the cell viability and proliferation were more efficacious under low frequency oscillatory magnetic stimulation, but there was no effect under thermal stimulation. In the same year, Ricotti et al. used a piezoelectrical trigger of glycol chitosan-boron nitride nanotubes with US transducer stimulation for modulation of F/G-actin [41]. This simulation used a US transducer to convert mechanical stress into electrical signals to investigate actin expression in human dermal fibroblasts. From these highlights of the timeline figure, the on-program trigger began to synergistically correspond to the variations of on-demand stimuli to improve therapeutic efficacy. As a result of this work in the past 10 years, these multiple-stimulus trigger DDS strategies have received dramatic progression due to rapid advanced bio-fabrication technologies.

### 2.2. Clinical Advancement

In the last 10 years, many novel drug carriers have been developed, but there has not been much completed clinical testing. Thus, there are 10 drug carriers for arthritis-related therapies that have completed the recruitment status of the United States Federal Government clinical trials identifiers (GCTI) [42]. There are four, two, two, one, and one drug carriers successfully invented for knee OA [43], RA [44], osteosarcoma [45], joint infections [46], and peri-implant bone therapies [47], respectively. For the primary tests, we observed that there were 5 pharmacokinetics tests out of 10 [48]. Moreover, the Western Ontario and McMaster Universities OA Index (WOMAC) is used as a self-administered health status for non-IA patients, such as knee OA patients, for pain management [49]. These dominant pharmacokinetics tests mean that the target concentration, distribution volume, and elimination clearance of API are the main considerations of clinical trials [50]. As a result, drug carriers with efficacy, programming release, and patient compliance features are the most preferable choices for scientists.

As shown in Table 1, drug carriers are generally classified into targeted biological-disease-modifying products and products resulting from the conjugation of hydrophilic and hydrophobic functional groups. The microRNA (miR)-146a [51] drug carrier is the targeted biological-disease-modifying product used to release tocilizumab [52,53]. MiR exhibits neurological disease dysfunction in the different stages of the central nervous system, which is not related to this topic and not further elaborated in this review. The other nine drug carriers are created from both functional group conjugations, such as multi-layer lipid in lyophilised formulation (TLC599), diclofenac etalhyaluronate (SI-613), corticosteroid fluticasone propionate with polyvinyl alcohol (PVA) coating (EP-104IAR), HA polynucleotides (PN) [54], tumour necrosis factor (TNF) inhibitor (adalimumab) [55,56], albumin-bound inhibitor with mammalian target of rapamycin (mTOR) (ABI-009) [57], sirolimus derivatives with mTOR inhibitor kinase (temsirolimus), active Ted compound in phosphate ester prodrug (tedizolid phosphate) [58], and platelet-rich fibrin (PRF) [59]. The hydrophilic functional groups are designed to carry API, such as dexamethasone (Dex) sodium phosphate [60], diclofenac [61,62], fluticasone propionate [63], PN [64,65], methotrexate (Mtx) [66,67], nivolumab [68,69], liposomal Dox [70,71], tedizolid (Ted) [72,73], and simvastatin [74,75], which cleave autonomously with endogenous stimuli. However, these inventions still have not solved the systemic toxicity problem due to their long-term uses. Therefore, more advanced inventions with on-program triggers are moving into clinical trials. As a result, this review elaborates on the inventions resulting from the composition of on-demand stimuli and on-program triggers.

## 3. Pathological Alterations for Endogenous Stimuli

Pathological alterations [96] are the alerts or signal transmissions caused by the activities of infectious bacterial diseases [97], such as Staphylococcus aureus (*S. aureus*) in most cases [98]. The bacterial diseases of OA and RA consume oxygen in the metabolism to produce enzymatic secretions, heat, and carbon dioxide. Therefore, redox, enzymes, and heat are three pathological alterations. The fourth is pH pathological alteration due to the mild acidity resulting from the excess of carbon dioxide in the tumour microenvironment (TME). In fact, the respiration metabolism above also produces electro-ions from the specific protonation of proteoglycan contents’ cations in this acidity TME, so the electro-ion is the fifth pathological alteration. As a result, there are five endogenous stimuli, namely, enzyme, redox, hyperthermia, pH, and electro-ions, used for the specific pathological alterations such as degradation enzyme, reactive oxygen species (ROS) disorders, temperature abnormalities, mild acidity, and electronegative potentials. Moreover, a drug carrier could be engineered with hydrophobic functional groups, such as oleic acid of amine and carbodiimide of carboxylic acid, to conjugate with hydrophilic API [99], which allows them to cleave autonomously due to endogenous stimulation. Both hydrophobic and hydrophilic conjugations increase the pharmacokinetics modulation after systemic drug administration [100].

### 3.1. Enzymatic Stimulus

Enzymatic stimulus is an API release mechanism induced by the matrix metalloproteinase (MMP) enzymatic secretions [101] of an enzymatic antigen to cleave the peptide (P)-bonds [102]. The rapid proliferation and metastasis of tumour cells disturbs the normal cell homeostasis, resulting in the enzymatic secretions being elevated to regulate the enzyme levels [103]. Therefore, the progression of OA and RA could be easily detected by intervening in enzymatic secretions. As a result, enzyme stimulus could be used as an enzymatic biosensor to detect this enzymatic secretion as a signal of pathological alteration to simulate API release in the synovial membranes.

The four most common types of enzymatic biosensors are endopeptidases of MMP-2 and MMP-9 [51], a disintegrin and metalloproteinase with thrombospondin motifs 5 (ADAMT5) proteases [104,105], and TNF-α pro-inflammatory cytokine [106]. These MMPs are proenzymes, secreted with calcium-dependent, zinc-containing endopeptidases in order to remodel tissue with various physiological or pathological processes. ADAMT5 is an enzyme secreted with multi-domain zinc endo-MMP, which is an anti-angiogenic and anti-cancer protein mediating proteolytic signalling processing independently. TNF-α stimulates MMP-13 and prostaglandin E2 production. This review focuses on the discussion of MMP-2 and MMP-9 enzymatic biosensors only. For instance, Joshi et al., (2018) used enzymatic biosensors of MMP-2 and MMP-9 in triglycerol monostearate (TG-18) hydrogel disassembly to investigate the release of triamcinolone acetonide (TA) in an arthritis flare-responsive DDS [107]. In addition, Li et al., (2019) studied the same enzymes using a different biosensor of transcription-transactivating protein (TAT) to release ANXA_1_ tripeptides (QAW) [108] or Dex in an RGD-MMP-TAT-QAW (RMTQ) drug carrier for two individual experiments [109]. Coincidentally, both studies showed similar findings of enzyme-stimulated inflammation efficacy and target-specified delivery. This QAW attenuates surgery-induced neuroinflammation and memory deficits through regulation of the NOD-, LRR-, and pyrin-domain-containing protein 3 (NLRP3) inflammasome.

### 3.2. Redox Stimulus

The ROS and glutathione (GSH) levels are in equilibrium or balancing in the normal and healthy cell microenvironment, but not in the TME. Therefore, this inequilibrium of ROS and GSH is used as a pathological alteration to design a redox stimulus for DDS. ROS produce oxygen gas, resulting in oxidative stress and lesion progression, but GSH fights the oxidative stress through an antioxidant system. Therefore, the relationship between ROS and GSH amounts is always in contrast. Even an excess of ROS will eliminate tumours, but more GSH will resist DDS efficacy. However, in the lesion or infectious condition, ROS is reduced, leading to a redox microenvironment. As a result, the redox stimulus could be designed using the variations of ROS decrement and GSH increment as a pathological alteration to simulate API release.

Redox biosensors have three common types, such as ROS, peroxide oxidation, and antioxidants. Their specified names are represented by hydroxyl radicals, poly(propylene sulphide) (PPS), and SOD or poly(2-ethyl-2-oxazoline) (PEtOx), respectively. For instance, Zhong et al., (2022) used SOD as a redox biosensor in a human serum albumin (HSA) drug carrier to release Mtx for RA therapy [110]. The significant findings were that SOD scavenged ROS and Mtx suppressed inflammation with reduced side effects. This result showed Mtx is a DMARD for chemotherapy, being successfully used as an anti-neoplastic agent in an immune-system suppressant.

### 3.3. Hyperthermia Stimulus

Hyperthermia stimulus is a pathological alteration of more than 37 °C, whereby a thermal sensitivity biosensor is induced to release API. Therefore, the biosensor should be reacted to after 37 °C because the human body is in TME, and the upper critical temperature should be less than 43 °C. As far as we know, extra heat is produced from all the other four stimuli. As a result, this hyperthermia stimulus is a necessary factor that should be integrated into other stimuli.

The most common types of hyperthermia biosensors are metal nanoparticles. For instance, Shen et al., (2022) used a different hyperthermia stimulus biosensor of poly(acrylonitrile-co-acrylamide)-polyethylene glycol (P(AN-co-AAm)-PEG) micelles to investigate the release profiles of tofacitinib (Tof) and ICAM-1 antibody (AI) [111]. The biosensor results showed that their particle sizes increased from less than 100 nm at 37 °C to more than 800 nm at 43 °C before disassembling. The significant findings showed that both API release profiles were approximately 40% at 37 °C and more than 80% at 43 °C within 48 h. This was better than the conventional biosensor because the detection range was hyperthermia, ranging from 37 °C to 43 °C.

### 3.4. pH Stimulus

Acidity or a low pH microenvironment is a growth factor of TME, one that can be used as a pathological alteration to design a pH stimulus for DDS. The mild acidity microenvironment begins with lactic acid or pyruvic acid production from the glycolysis metabolic process. This process occurs in the TME because the glucose oxidation process is retarded by limited oxygen supply. Then, the normal cell respiratory metabolism without glucose oxidation is stopped, and the tumour cell glycolysis metabolism is boosted. As a result, the pH stimulus could be designed using the feature of mild acidity TME as a pathological alteration to simulate API release.

The four most common types of pH biosensors are chitosan (Chi), amide functional groups, dextran derivatives, and meso-2,3-dimercaptosuccinic acid (DMSA). For instance, Lee et al., (2015) used Chi as a standard pH biosensor to stimulate and release tobramycin in a poly(acrylic acid)/Chi drug carrier [112]. The significant findings showed tobramycin successfully reduced *S. aureus* bacteria activity. Furthermore, Wang et al., (2022) used acetalated dextran (AcDEX) as a biosensor of pH stimulus DDS to release Mtx in RAW264.7 and its LPS-activated cells for RA therapy [113]. The significant findings showed that Mtx regulated the JAK-STAT pathways between protein cells and processing cells, resulting in potent pharmacokinetic and pharmacodynamic profiles.

### 3.5. Electro-Ion Stimulus

Electro-ion stimulus is a pathological alteration of the specific protonation of charge segregation in membrane potential induced by the electropositive ions from tumour cells, resulting in endogenous electric fields [114]. This charge segregation is the electro-ion gradient between normal cells ranging from −90 mV to −40 mV [115] and tumour cells of −11 mV [116]. This is due to the healthy proteoglycan contents with a net electronegative ion, but not in tumour cells. This electro-ion is synchronised to produce signals, radical outcomes, and Ca^+2^ channels that are very important for the proteoglycan content streaming potential in repairing skeletal tissue regeneration. As a result, the electro-ion stimulus could be designed by using the signals, radicals, and ions as pathological alterations to simulate API release.

The three most common types of electro-ion biosensors are metal/metallic organic frameworks (MOF), conductive polymers, and bioactive ligands/linkers. For instance, Bátai et al., (2022) used transient receptor potential cation channel (TRP) A1 as an electro-ion biosensor to stimulate dimethyl trisulphide release in K/BxN serum-transfer arthritis for RA immunological mechanisms [117]. TRPA1 is a cationic channel that is activated by electrophilic dimethyl trisulphide chemicals, resulting in a peak current of +50 mV. The significant findings showed that TRPA1 had nociceptor neurons with anti-inflammatory effects by developing milder collagen deposition in the inflamed joints.

**Figure 2:**
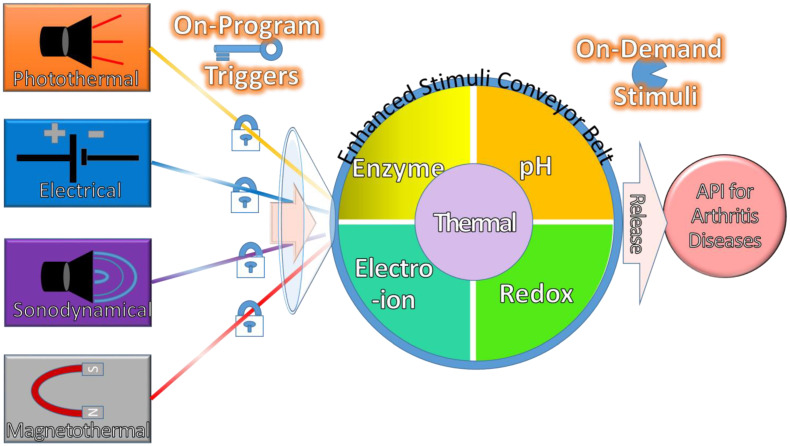
The mechanisms of multiple on-program triggers are enhanced with multiple on-demand stimuli to release API for arthritis diseases.

In conclusion, there are five stimuli used to detect the pathological alterations, namely, redox, enzyme, hyperthermia, pH, and electro-ions. However, extra heat or hyperthermia occurs in all stimuli, so it should not be an independent endogenous stimulus. This thermal factor should be integrated into other stimuli or triggers, as shown in the centre of Figure 2. Therefore, there are four independent endogenous stimuli, namely, enzyme, redox, pH, and electro-ions that are used to compose with the four dependent triggers, as shown in the mechanism description in Figure 2. These four endogenous stimuli are generally detected by their biosensors consisting of enzymatic antigens, ROS decrements, hydroxyl functional groups, and cationic channels. Generally, there are four, three, four, and three most common types of biosensors for enzymes, redox, pH, and electro-ions, respectively. As a result, these biosensors are being stimulated by their respective pathological alterations to release API on demand for arthritis disease therapy.

## 4. Exogenous Triggers with Endogenous Stimuli

Traditionally, there are six exogenous triggers in DDS, such as NIR, magnetic, electric, acoustic, mechanical, and piezoelectricity. Recently, these six have been reconstructed into five exogenous triggers, such as photothermal, magnetothermal, electrical, sonodynamical, and piezoelectricity triggers, respectively. The thermal term is added to NIR and magnetic triggers because heat is always produced during the triggering process. Furthermore, the dynamical term is added to the acoustic trigger because dynamic force is always produced during the triggering process. However, we think the piezoelectricity trigger is an overlapping of the dynamic force in the sonodynamical trigger and the electro-ion endogenous stimulus. In addition, the advanced DDS always emphasises deeper target penetration nowadays by using sono acoustics. Therefore, this should be an independent sonodynamical trigger that is used to compose with an independent endogenous stimulus. As a result, there are four exogenous triggers in DDS, namely, photothermal, magnetothermal, sonodynamical, and electrical triggers.

These composed mechanisms of multiple on-program triggers are enhanced with multiple on-demand stimuli to release API for arthritis diseases, as shown in Figure 2. These four exogenous triggers are independently composed of either one, two, or more endogenous stimuli for the on-demand pathological alterations in order to manipulate the release behaviour of API for therapeutic efficacy and efficiency. Thus, these API could be administered into the body by the on-program exogenous triggers at predetermined time intervals for future endogenous stimuli. This programming therapy allows API to be stimulated on demand by endogenous stimuli at any time to kill the disease. Ideally, API is delivered on demand at the right time and releases the right dose by remote control at the desired time into the lesion site to improve efficacy with minimal side effects. As a result, a smart DDS with targeted delivery and controlled release could be developed by exploiting the characteristics of endogenous and exogenous stimulation properties.

### 4.1. Photothermal Triggers

Photothermal trigger is formerly known as a near-infrared (NIR) stimulus because it usually uses NIR light beams with heat to stimulate API. NIR wavelengths from 450 to 980 nm are used to penetrate into a specified deep-seated tumour target site to release API precisely. The biosensors, such as gold (Au), copper, carbon dots, graphene oxide (GO), and molybdenum disulphide (MoS_2_), are used to accelerate the decatalysation of drug carriers.

The photothermal triggers with additional stimuli in drug carriers are elaborated with biosensors and remarks, as shown in Table 2. For instance, Xue et al., (2022) used mesoporous copper sulphide (CuS) NP as a photothermal biosensor in a CuS@neutrophil-erythrocyte (NR) drug carrier to release Dex for anti-IA therapy [118]. Dex has the role of a glucocorticoid receptor agonist and an anti-inflammatory medication. The significant findings showed that there were high levels of efficiency, target reachability, and long-circulated releases. Another special biosensor of up-conversion luminescent NP (UCNP) was used by Zhang et al., (2013) [119], which reacted to NIR due to the trivalent ytterbium (Yb^3+^) ion from UCNP (NaYF_4_:Yb^3+^/Er^3+^). In this study, *N*-isopropylacrylamide-*co*-methacrylic acid (NIPAMAA) and UCNP were added to an UCNP@SiO_2_/Poly(NIPAMAA) (PIPAMAA) drug carrier as the pH stimulus and photothermal trigger biosensors, respectively. The significant findings showed that this release of Dox increased with higher temperatures and lower pH values. In addition, Wen et al., (2018) investigated the pH stimulus and photothermal trigger of Dox release in a poly(β-amino ester) (PBAE)@UCNP drug carrier using another UCNP and PBAE as biosensors, respectively [120]. Their significant findings showed enhanced PBAE protonation in lysosomes to release Dox.

#### 4.1.1. Photothermal Triggers Added Magnetic-Aid Targetor or Photoacoustic Detection

Traditionally, the magnetic NP has been found to be the best for deeper and specified target delivery. Therefore, researchers and scientists prefer to add IONP as a magnetic-aid targetor to photothermal triggers and other stimuli, as shown in Table 3. For instance, Wang et al., (2013) used azobenzene derivatives (Azo) as a photothermal biosensor with the assistance of magnetic mesoporous silica (MMS) as a magnetic-aid targetor in an Azo-MMS drug carrier to release ibuprofen [121]. In another study involving IONP magnetic-aid targetor in the release of nitric oxide (NO) and partial thromboplastin time (PTT), Yu et al., (2020) used ruthenium (Ru) as a photothermal biosensor in a polydopamine (PDA)/IONP/Ru-NO/folic acid (FA) drug carrier [122]. The significant findings with another special biosensor of Ru showed enhanced antitumor efficacy with the assistance of a magnetic-aid targetor. Recently, a photothermal trigger was assisted by photoacoustic (PA) detection for better pharmacokinetic monitoring. PA detection is a biomedical imaging modality that uses NIR absorption and US emission. For instance, Zhao et al., (2019) used MoS_2_ as a photothermal biosensor with the assistance of PA detection in a MoS_2_/Chi drug carrier to release Dex [123]. The significant findings revealed the TNF-α and IL-1β secretions were reduced and cartilage erosion was delayed. Kim et al., (2022) used Au as photothermal trigger with a PA biosensor of DNA aptamers (DNAA) to release dimethyl sulfoxide (DMSO) for human MMP-9 detection [124]. The significant findings revealed that this biosensor of the double-stranded AuNP-1 (Au1) and single-stranded AuNP-2 (Au2) aptamers was selective and sensitive to human MMP-9 detection.

#### 4.1.2. Photothermal Triggers Added Magnetic-Aid Targetor with pH or Redox Stimulus

In another three studies, this magnetic-aid targetor assisted the photothermal trigger by adding pH and redox stimuli into drug carriers with biosensors, as shown in Table 4. For instance, Deng et al., (2016) used Chi and GO as biosensors of Dox in an alginate (Alg)/Chi/IONP@GO/HA drug carrier for the pH stimulus and photothermal trigger, respectively [125]. Their significant findings showed a synchronised effect of thermal and chemotherapy with magnetic-aid targetor assistance. Oh et al., (2017) used DMSA and IONP as biosensors in an IONP/DMSA drug carrier for the pH stimulus and photothermal trigger of Dox release, respectively [126]. The findings showed IONP could be used as a targetor and also a photothermal trigger because Fe^3+^ temperature was excellently induced by NIR. Lastly, Jia et al., (2020) used PDA as a photothermal trigger of Dox release in an IONP/SiO_2_@PDA drug carrier into bovine serum albumin (BSA) [127]. Their significant discovery demonstrated PDA increased hydroxyl radical production in the Fenton reaction and found efficacy in thermal and chemodynamic therapy. As a result, this hydroxyl radical from PDA could be used as a redox stimulus for photothermal triggering, potentially improving osteosarcoma therapy efficacy.

**Figure 3:**
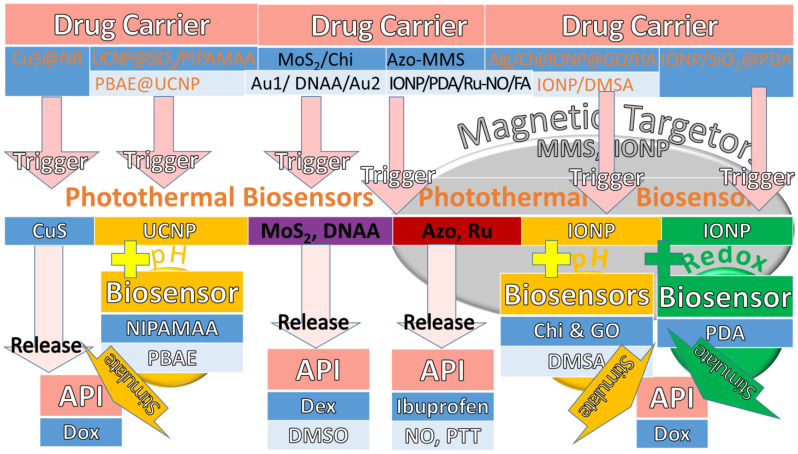
The photothermal triggers with magnetic targetors add redox and pH biosensors in drug carriers to release API. The first and second flows from drug carriers to API are shown in dark blue and light blue, respectively.

In conclusion, the photothermal triggers with magnetic targetors add redox and pH biosensors in drug carriers to release API, as shown in Figure 3. The different colours of cells are used to categorise the first and second flows from drug carriers to API, as shown in dark blue and light blue, respectively. The most common biosensors for photothermal triggers are metal ingredients such as IONP and magnetic NPs such as MMS, which are used as magnetic-aid targetors. Some photothermal trigger tests use MoS_2_ and DNAA as biosensors and PA detectors as well. Moreover, the photothermal biosensors of CuS, Azo, PDA, and Ru are found to have high efficiency, target reachability, and long-circulated releases. Some use photothermal biosensors such as UCNP and GO to compose with pH endogenous stimuli such as NIPAMAA, PBAE, Chi, and DMSA to find a good release profile for Dox. Furthermore, some use PDA as a redox stimulus to compose into photothermal biosensors to release API for therapeutic efficacy in thermal and chemodynamic therapy.

### 4.2. Magnetothermal Triggers

Magnetothermal trigger is a reverse orientation of the electromagnetic or superparamagnetic field on magnetic NP (MNP) [128], resulting in heat and API releases at a target site [129]. This superparamagnetism from an external electromagnetic field is used to generate magnetic hysteresis on MNP, such as iron oxide NP (IONP) of magnetite (Fe_3_O_4_) and maghemite (γFe_2_O_3_) [130]. The MNP sizes include more than 100 nm, less than 100 nm, and more than 20 nm, which are used in superparamagnetic heat for hysteresis loss [131], Neel motions [132], and Brownian motions [133], respectively. For instance, Su et al. used IONP as a magnetic biosensor in a drug carrier with lactoferrin and perfluorohexane for the magnetothermal trigger of paclitaxel (Ptx) release for tumour therapy [134]. The heat from superparamagnetism induced perfluorohexane gasification, resulting in Ptx release into tumour cells. As a result, this continuous magnetic heat led to hyperthermia and paclitaxel delivery that eradicated tumour cells with this effective chemo-thermotherapy.

#### Magnetothermal Triggers with Redox and pH Stimuli

In order to have a greater variety of endogenous stimuli, the magnetothermal triggers of API release are enhanced by either redox, pH, or both redox and pH stimuli in drug carriers, as shown in Table 5. This table elaborates how the IONP biosensor is used for magnetothermal triggering that adds additional stimuli and biosensors in drug carriers, with remarks listed. For instance, Modak et al., (2020) investigated the redox stimulus of PPS and the magnetothermal trigger of IONP using a drug carrier of PEG-*block*-PPS/IONP to release Dox or camptothecin for different tests of multicargo intracellular DDS [135]. The findings showed that PPS was oxidised by ROS and IONP was magnetised to release heat for API inductions. Furthermore, Jafari et al., (2021) added Chi to IONP as biosensors of pH stimulus and magnetothermal trigger of sunitinib release, respectively, in magnetic hydroxyl propyl methyl cellulose (mHPMC)/IONP-Chi drug carrier [136]. The significant findings showed that Chi released sunitinib efficiently at pH 4.5. Another study was conducted by Soleimani et al., (2021), who composed the pH and redox stimuli into PEtOx for the magnetothermal trigger of Dox release for chemo-hyperthermia cancer therapy [137]. The significant findings were that β-cyclodextrin/*g*-(PEtOx)_7_/IONP drug carrier showed therapeutic effects on PEtOx with pH 7.1, GSH, and IONP ionised tumour cells at about pH 5 for pH, redox stimuli, and magnetothermal trigger, respectively.

**Figure 4:**
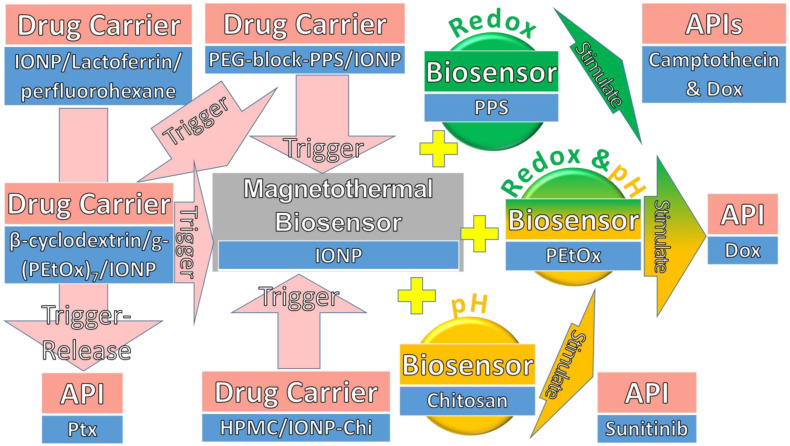
The magnetothermal triggers added with either redox, pH, or both stimuli into biosensors in drug carriers to release API.

In conclusion, the magnetothermal triggers add either redox, pH, or both stimuli into biosensors in drug carriers to release API, as shown in Figure 4. As usual, the magnetothermal biosensor in a drug carrier is mostly IONP. Chemo-thermotherapy was discovered to be effective when using an IONP magnetothermal trigger to release Ptx into tumour cell eradication. Another magnetothermal trigger composed of PPS as a redox biosensor in a multicargo intracellular DDS was discovered to be effective for releasing Dox or camptothecin. Moreover, this magnetothermal trigger also composed of Chi as a pH biosensor and was found to efficiently release sunitinib at pH 4.5. Lastly, this magnetothermal trigger composed both pH and redox stimuli into a biosensor of PEtOx was found to efficiently release Dox for chemo-hyperthermia cancer therapy.

### 4.3. Sonodynamical Triggers

Sonodynamical trigger is formerly known as an acoustic stimulus because it usually uses high-intensity, US-focused sound waves to generate stable cavitation that oscillates chemical sonosensitisers around to release API. In recent formulations, the sonodynamical trigger uses 1 MHz US pulses to generate dynamical force to release API until about 10 cm depth. The US oscillation is used to activate the cavitation of biosensors, such as NP of TiO_2_ and liposome, porphyrin, microbubbles, and IR780 dye.

The sonodynamical triggers with biosensors to release API in drug carriers are elaborated with remarks, as shown in Table 6. For instance, Jing et al., (2011) used US to crack the TiO_2_ shell and IONP in a TiO_2_/IONP/PEG-graphene-quantum dot (QD) drug carrier to release Ptx and magnetic targeting, respectively [138]. The significant findings showed the release profile being controlled by applying US duration. Furthermore, Wang et al., (2018) used a standard sonodynamical biosensor of porphyrin in a drug carrier made of porphyrin-phospholipid (PP) to release Dox [139]. The significant findings showed enhanced local delivery and tumour suppression. In the advanced lipid technology, the liposomal NP becomes a favourable biosensor in DDS. For instance, Zhou et al., (2019) used asparagine-glycine-arginine peptide (NGR) liposome as a sonodynamical biosensor in a 1,2-distearoyl-sn-glycero-3-phosphoethanolamine (DSPE)-PEG_2k_/NGR drug carrier to release Dox [140]. The significant findings showed the lipid bilayer was triggered to breakdown for Dox release in antitumor effects. Recently, Dwivedi et al., (2020) used a traditional sonodynamical biosensor of about 4 μm perfluorocarbon gas microbubble (PFCMB) in an IONP/PEG/PFCMB drug carrier to release Dox for anticancer therapy [141]. Their significant findings were that magneto-liposome of PEG was added to assist in deeper site targeting and precise delivery.

#### Sonodynamical Triggers with Redox and Electro-Ion Stimuli

Much research has shown that US irradiation produces ROS, but not many studies used both as sonodynamical and redox biosensors. Some examples of sonodynamical biosensors have had redox and electro-ion stimuli added to trigger API in drug carriers, as shown in Table 7. For instance, Wu et al., (2019) used IR780 dye and thioketal linkers (TL) as sonodynamical and redox biosensors, respectively, in a 1,2-distearoyl-sn-glycero-3-phosphoethanolamine (DSPE)-PEG_2k_-NH_2_/IR780/TL drug carrier [142]. The significant findings showed ROS was simulated under US, which cleaved TL to release Ptx for tumour growth inhibition and apoptosis. Furthermore, Kim et al., (2011) used TiO_2_ and phenylboronic ester (PBE) as sonodynamical and redox biosensors, respectively, in a TiO_2_-PBE drug carrier [143]. The significant findings showed US cracked TiO_2_ and simulated ROS to cleave PBE and release Dox for high local accumulation and tumour growth inhibition. Moreover, the sonodynamical trigger adds an electro-ion stimulus to generate an US piezoelectric mechanism, which converts the dynamical force to electro-ion stimuli to release API. For instance, Liang et al., (2019) used tetra-(4-aminophenyl) porphyrin (TAPP) for sonodynamical stimulus and CuS and Pt for the electro-ion and photothermal stimuli in a Pt-CuS/TAPP drug carrier for the sonodynamical and photothermal cancer therapies [144]. In addition to CuS, Pt possesses nanozyme activity for catalysing hydrogen peroxide (H_2_O_2_). The significant findings showed that H_2_O_2_ produced O_2_ for ROS in tumour hypoxia and cell apoptosis therapies.

**Figure 5:**
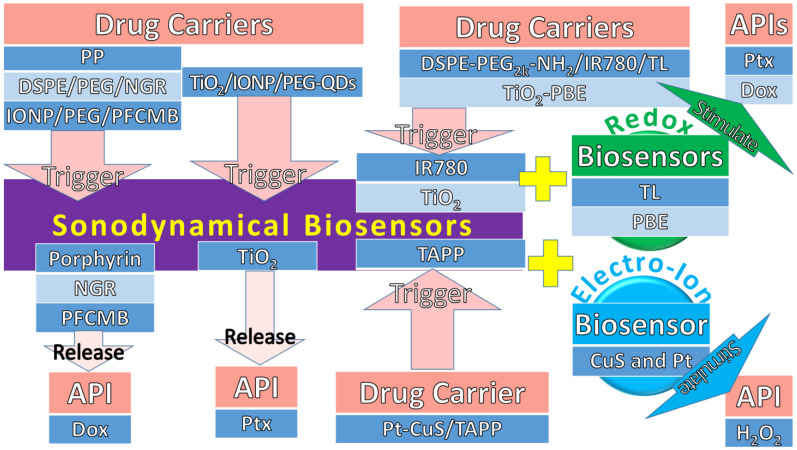
The sonodynamical trigger with either redox or electro-ion stimuli added into their biosensors in drug carriers to release API. The flows from drug carriers to API are represented by the first and third cells, and second cells, as shown in dark blue and light blue, respectively.

In conclusion, the sonodynamical triggers add either redox or electro-ions into biosensors for drug carriers to release API, as shown in Figure 5. In order to differentiate the flows from drug carriers to API, the first and third cells, and second cells are highlighted in dark blue and light blue, respectively. A TiO_2_ shell was used as a sonodynamical biosensor and showed a controlled release profile of Ptx. The other three sonodynamical biosensors, namely, porphyrin, NGR, and PFCMB, were used to release Dox, showing enhanced local delivery, anti-osteosarcoma, and itssuppression. Another two sonodynamical triggers of IR780 dye and TiO_2_ with added redox stimuli of TL and PBE into biosensors showed US induced ROS to release Ptx and Dox, respectively, for osteosarcomagrowth inhibition. Lastly, a sonodynamical trigger of TAPP added CuS and Pt for the electro-ion and redox stimuli for osteosarcomahypoxia and cell apoptosis therapies.

### 4.4. Electrical Triggers

Traditionally, the electrical trigger uses metal NP mediating dipole interactions as a biosensor, which prevails in surpassing the molecular motion of Van der Waals or repulsive electrostatic forces, resulting in API release to a target site. Recently, this electrical trigger of about 10 mA of current and 100 Hz bipolar electric pulses [145] was used to unseal the drug reservoirs to release API. Due to the recent nanotechnology progress of the semiconductor industry, the development of electrical biosensors has been stimulated by microchips, such as micro electro-mechanical systems (MEMS) [146]. This MEMS is transformed into drug reservoirs made of electrolyte-impermeable substrate that can be stored by sealing off the membranes and triggering them to devoid or disintegrate to release API.

#### 4.4.1. Electrical Triggers with Metal-Organic Frameworks

Electrical biosensors have traditionally been composited using MOF technology [147] because it is cost effective and reduces the hazardous by-products of metal or metallic substances. The electrical performance of CuZn and Cu being used as electrical biosensors to trigger API release in drug carriers was highlighted, as shown in Table 8. Recently, well-known semiconductor materials such as CuZn have become a popular choice as electrical biosensors for API release [148]. Moreover, CuZn has higher membrane stress to provide mechanical strength for the articular cartilage scaffold [149]. For instance, Forero et al., (2017) made a gelatin (G)/CuZn/Chi drug carrier with CuZn as an electrical biosensor to trigger hydroxyapatite (HAp) release [150]. The significant performance showed that anti-bacterial activity increased, but osteoprogenitor cells were not cytotoxic. Even though this finding showed that it is suitable for arthritis drug carriers, the electrical performance was not measured. Furthermore, Wang et al., (2018) measured the electrical performance of a Cu electrical biosensor using differential pulse voltammetry (DPV) in an Au@Cu drug carrier [151] made by MOF technology. The significant findings found that it was highly sensitive to the miR-155 release at its detection limit of 0.35 fM and recorded a current response change of 25 µA. From here, the measurement of electrical biosensor performance uses the current response changes of microamperes compared to the miniamperes before that. In addition, Gharehdaghi et al., (2022) measured the Cu MOF electrical biosensor by using two drug carriers of benzene 1,3,5-tricarboxylate (BTC)_2_/Cu_3_/IONP and GO/Cu/tetrakis (4-carboxyphenyl) porphyrin (TCPP) to trigger Dox release [152]. Despite the electrical performance of a Cu MOF electrical biosensor, their significant findings were the acidic responsive at pH 5, which demonstrated the API adsorptions of 40.5 wt % and 45.7 wt % and release of 85.5% and 98.9%, respectively. As a result, the GO/Cu/TCPP drug carrier had better adsorption and release than the (BTC)_2_/Cu_3_/IONP drug carrier at pH 5.

This acidic condition study is an important consideration because the electrolyse process most of the time will produce acidic products. For example, the glucose oxidation process and the acid chemical reaction were previously discussed in our paper, as shown in Equations (1) and (2), respectively [148].

(1) $$C_{6}H_{12}O_{6}+6O_{2}\to 6{\mathrm{CO}}_{2}+6H_{2}O+\mathrm{Energy}$$

(2) $${\mathrm{CO}}_{2}+H_{2}O\to {\mathrm{HCO}}_{3}^{-}+H^{+}$$

where C_6_H_12_O_6_, O_2_, CO_2_, H_2_O, HCO_3_^−^, and H^+^ are glucose, oxygen, carbon dioxide, water, bicarbonate, and hydrogen ions, respectively. As we know, the tumour grows and proliferates in acidic conditions. Therefore, a drug carrier with a higher adsorption and release of API is better to inhibit tumour growth and proliferation.

#### 4.4.2. Electrical Triggers with Conductive Polymers

Conductive polymers are organic polymers that conduct electricity [153] and may have metallic conductivity such as semiconductors. They are used as biosensors for electrical triggers such as the derivatives of polypyrrole, polyaniline (PANI), polyacrylamide, and polymethacrylic acid, as shown in Table 9. For instance, Atoufi et al., (2017) used aniline tetramer (AT) as an electrical biosensor in an agarose (Agr)/Alg/AT drug carrier to release Dex [154]. The significant findings showed enhanced cell viability and proliferation in neuroregeneration medicine. Furthermore, Yu et al., (2020) used iron-oxide-nanocube clusters (IONC) as an electrical biosensor to release indoleamine 2,3-dioxygenase inhibitors (IDOi) in an IDOi/IONC drug carrier [155]. The significant findings showed that the magnetic IONP can be used as an electrical biosensor to enhance cell damage by the synergistic immuno-ablation cancer therapy. Mohapatra et al., (2020) used PEG dimethacrylate (PEGDMA) as an electrical biosensor to release vancomycin (Vcm) in a Chi/IONP/PEGDMA drug carrier [145]. The significant findings showed controllable electrical stimulation of DDS with the assistance of the magnetic iron-oxide NP in site targeting. Lastly, Patil et al., (2020) used pullulan (Plt)/PVA as an electrical biosensor to release rivastigmine tartarate (RT) in a Plt/PVA/polyAAm (PAA) drug carrier for transdermal DDS efficiency [156]. Even though RT is used for Alzheimer’s disease, it can be replaced with API used for RA, such as capsaicin, salicylates, counterirritants, and anaesthetics in future research.

**Figure 6:**
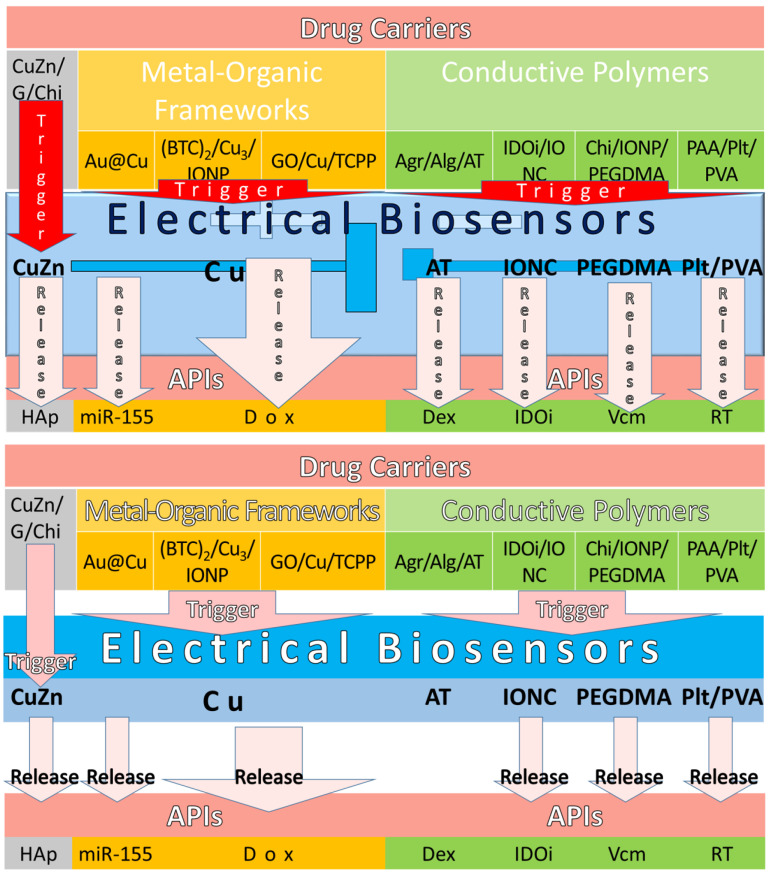
The electrical biosensors are triggered to release API in drug carriers that are made of MOFs and conductive polymers.

In conclusion, the electrical biosensors are triggered to release API in drug carriers that are made of MOFs and conductive polymers, as shown in Figure 6. The electrical biosensors made of CuZn and AT to release Hap and Dex, respectively, were found to be suitable for arthritis drug carriers. The Cu MOF electrical biosensors were discovered to have highly sensitive electrical performance with a 0.35 fM detection limit and a 25 µA current response change. Moreover, Cu MOF electrical biosensors also found higher API adsorption and release in acidic conditions of pH 5. Furthermore, the electrical biosensors such as IONC, PEGDMA, and Plt/PVA were found to be synergistically controlled and efficient in DDS.

#### 4.4.3. Electrical Trigger with Enzymatic Stimulus

An electrical biosensor [157] is a biochemical signal-detector generally constructed by a bio-recognition receptor, transducer, and signal processing system. An enzymatic biosensor [146] uses an enzyme as a receptor, which catalyses with a specific analyte without adding any reagent. In the enzymatic stimulus mechanism, the specific analyte such as an enzyme antigen is used as a transducer for enzymatic cleavage to release API as a signal processing system. For normal voltammetric biosensor detection, there are gelatinase enzymes, either MMP-2 or MMP-9 [158], or both, that are used in OA and RA therapies. These enzymes disrupt the basal membrane barrier, resulting in the growth, angiogenesis, and metastasis of tumours. Therefore, the relationship between these enzymes and bacteria is used to create an enzyme-based biosensor. Both biochemical reactions of antigen–antibody and bacteria–enzyme are established due to the interaction of antibody against bacteria, resulting in a specific antigen–enzyme interaction.

In the last four years, the electrical triggers added MMP enzymatic biosensors and their cleavage sites in drug carriers with test remarks, as shown in Table 10. The results found four cleavage site tests on MMP-2 and MMP-9 each, and one on both enzymes. For instance, Wang et al., (2019), Li et al., (2020), and Cheng et al., (2021) discovered the MMP-2 cleavage sites located between glycine (Gly) and valine (Val) in three different electrochemical tests using drug carriers of carbon sphere (CS)-Au-Pb/P-NH_2_/PANI [159], DNA-P/rDNA [160], and P-mercaptoundecanoic acid (PMUA)/cucurbit[8]uril (CB8)/Ag/P2 [161], respectively. These results showed that the Gly-Val P-bond could be used in a biosensor to detect the MMP-2 enzyme. Thus, this could be a guideline for scientists to further investigate a biosensor, including the acrylamide sensitivity in P-bonds, a smaller size, and a simplified P-bond. After that, Fan et al., (2022) discovered the MMP-2 cleavage sites in another electrochemical test that was located in sulfhydryl P for a drug carrier of nitrogen-doped graphene quantum dots (NGQDs)/sulfhydryl P/Ru@SiO_2_ [157]. As a result, this specific reporter indicated that sodium dodecyl sulphate P-bond and graphene quantum dots could also be used in a biosensor to detect the MMP-2 enzyme. Furthermore, Park et al., (2019), Su et al., (2019), Liu et al., (2021), and Nisiewicz et al., (2022) discovered MMP-9 cleavage sites located between leucine-enkephalin (Leu) and methionine-enkephalin (Met) or between Gly and either Leu or Met using drug carriers of MoS_2_/Si/Amyloid β_1–42_ (Aβ_1–42_) [162], Fe_3_O_4_/Ir[(2-phenylpyridine)_2_(His-rich P)]CF_3_SO_3_ (Ir(III)His-P) [163], SiO_2_/rhodamine b/P-pentynoic acid (P-P) [164,165], and Au/cysteamine hydrochloride (CSH)/Gly-Met/ferrocene [166], respectively. The field-effect transistor (FET), fluorescence resonance energy transfer (FRET) [165], and DPV tests were used to discover these MMP-9 cleavage sites, which are some of the electrochemical tests. As a result, Gly, Leu, and Met P-bonds showed their potential to be used in a biosensor to detect the MMP-9 enzyme. In conclusion, these findings are vitally important for scientists to design a better biosensor.

**Figure 7:**
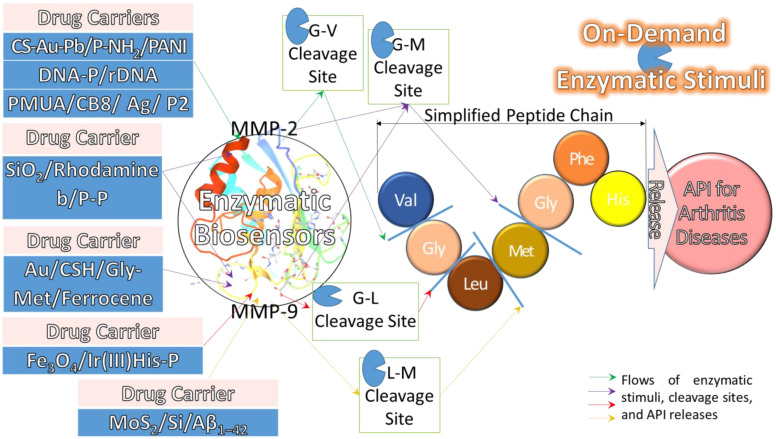
The enzymatic stimulation mechanisms of biosensors in drug carrier use MMP-2 and MMP-9 [158,167] to cleave peptide sites for API release in arthritis treatment. Reprinted with permission.

In conclusion, the mechanism P-sites are cleaved by enzymatic stimuli on their biosensors of MMP-2 and MMP-9 in drug carriers to release API for arthritis diseases, as those shown in Figure 7. In the enzymatic stimuli, the specific analytes of enzyme antigens, such as MMP-2 and MMP-9, are used to cleave for the release of API in OA and RA therapies. For MMP-2, three tests showed the cleavage P-sites located between Gly and Val. For MMP-9, three tests had the different cleavage P-sites located between Leu and Met, as well as between Gly and either Leu or Met. The cleavage P-sites of Gly-Val, Leu-Met, Gly-Leu, and Gly-Met are vitally important for scientists to design a better enzymatic biosensor.

## 5. Conclusions

For these multiple compositions between on-program triggers and on-demand stimuli, we proposed a hyperthermia stimulus be included in triggers because it is a common pathological alteration that constantly occurs. Therefore, these mechanisms become four independent exogenous triggers, namely, photothermal, magnetothermal, sonodynamical, and electrical, composed of four dependent endogenous stimuli, such as enzymatic stimulus, redox, pH, and electro-ion. The biosensors for these four stimuli are formulated to correspond to the four pathological alterations, namely, enzymatic antigens, ROS decrements, hydroxyl functional groups, and cationic channels. Generally, the most common types of biosensors for arthritis disease therapy are four, three, four, and three for enzymatic, redox, pH, and electro-ion stimuli, respectively. The magnetothermal biosensors, mostly IONP, could be used as photothermal biosensors and for magnetic targeted delivery. These biosensors can be added either to PPS as redox, Chi as pH, or PEtOx as both previous stimuli for DDS. Moreover, the photothermal biosensors are CuS, Azo, PDA, and Ru, as well as an additional PA detector function, such as MoS_2_ and DNAA. Some photothermal triggers of UCNP and GO were added either to PDA as a redox stimulus or to NIPAMAA, PBAE, Chi, and DMSA as pH stimuli. The sonodynamical biosensors are porphyrin, NGR, and PFCMB. Some biosensors of TiO_2_ and IR780 dye were added to TL and PBE as redox stimuli. Another TAPP biosensor was added, this time with CuS and Pt as electro-ion stimuli. Lastly, the electrical biosensors made of either MOFs such as IONC, CuZn, and Cu or conductive polymers such as AT, PEGDMA, and Plt/PVA were found to have highly sensitive electroperformance, higher API adsorption and release in pH 5 conditions, and synergistically efficient control in DDS. These electrical biosensors added enzymatic stimuli such as MMP-2 and MMP-9 that triggered them to cleave P sites for API releases. The results found three sites at Gly-Val for MMP-2 and the other three sites at Leu-Met, Gly-Leu, and Gly-Met for MMP-9. This composition of on-program triggers and on-demand stimuli allows for API therapy with advanced programming at any desired time to be delivered and released at the specified sites and the requisite doses, respectively.

## Figures and Tables

**Table 1:** Drug carriers for arthritis therapy, API, GCTI and year completed, and primary tests.

Drug Carrier	Arthritis Therapy	API	GCT I, Year	Primary Test	Refs.
TLC599 (BioSeizer^®^)	Knee OA	Dex sodium phosphate	NCT03754049, 2022	Pharmacokinetics	[76,77]
SI-613	Knee OA	Diclofenac (Voltaren^®^)	NCT03209362, 2021	WOMAC	[78,79]
EP-104IAR	Knee OA	Fluticasone propionate	NCT02609126, 2021	Pharmacokinetics	[80,81]
HA-PN (Condrotide^®^ Plus)	Knee OA	PN	NCT02417610, 2017	WOMAC	[82,83]
miR-146a	RA	Tocilizumab (Actemra^®^)	NCT03149796, 2017	miR expressions	[84,85]
Adalimumab (Humira^®^)	RA	Mtx	NCT01185288, 2014	Pharmacokinetics	[86,87]
ABI-009	Osteosarcoma	Nivolumab (Opdivo^®^)	NCT03190174, 2021	Pharmacokinetics	[88,89]
Temsirolimus (Torisel^®^)	Osteosarcoma	Liposomal Dox (Doxil^®^)	NCT00949325, 2019	Pharmacokinetics	[90,91]
Ted phosphate (Sivextro^®^)	Joint infections	Ted	NCT03378427, 2021	Immuno-compromised	[92,93]
PRF	Peri-implant bone	Simvastatin (Zocor^®^)	NCT05008068, 2021	Bone regeneration	[94,95]

**Table 2:** Photothermal triggers added to pH stimulus biosensors in drug carriers with remarks listed.

Biosensor	pH Stimulus	API	Drug Carrier	Remarks	Ref.
CuS	-	Dex	CuS@NR	High efficiency, target reachability, and long-circulated releases	[118]
UCNP	NIPAMAA	Dox	UCNP@SiO_2_/PIPAMAA	Release increased with higher temperature and lower pH values	[119]
UCNP	PBAE	Dox	PBAE@UCNP	Enhanced PBAE protonation in lysosomes to release Dox	[120]

**Table 3:** Photothermal triggers with the assistance of magnetic-aid targetors or PA detections in drug carriers with biosensors, with remarks listed.

Trigger	Assistance	Biosensor	API	Drug Carrier	Remarks	Ref.
Azo	magnetic	MMS	Ibuprofen	Azo-MMS	Used magnetic-aid targetor	[121]
Ru	magnetic	IONP	NO, PTT	IONP/PDA/Ru-NO/FA	Enhanced antitumor efficacy with targetor	[122]
MoS_2_	PA	MoS_2_	Dex	MoS_2_/Chi	Reduced cartilage erosion, TNF-α, and IL-1β secretions	[123]
Au	PA	DNAA	DMSO	Au1/DNAA/Au2	Selective and sensitive detection of human MMP-9	[124]

**Table 4:** Photothermal triggers added a magnetic-aid targetor with pH or redox stimuli in drug carriers with biosensors, with remarks listed.

Stimuli	Biosensor	API	Drug Carrier	Remarks	Ref.
pH	Chi	Dox	Alg/Chi/IONP@ GO/HA	Synchronised effect of thermal and chemotherapy	[125]
pH	DMSA	Dox	IONP/DMSA	NIR induced Fe^3+^ temperature excellently	[126]
Redox	PDA	Dox	IONP/SiO_2_@PDA	Additional redox stimulus from hydroxyl radicals	[127]

**Table 5:** Magnetothermal triggers with added redox and pH stimuli in drug carriers with biosensors, with remarks listed.

Stimuli	Biosensor	API	Drug Carrier	Remarks	Ref.
Redox	PPS	Dox, camptothecin	PEG-*block*-PPS/IONP	Heat trigger for API inductions in multicargo intracellular DDS	[135]
pH	Chitosan	Sunitinib	HPMC/IONP-Chi	Remote release of sunitinib efficiently at pH 4.5	[136]
Redox, pH	PEtOx	Dox	β-cyclodextrin/*g*-(PEtOx)_7_/IONP	Ionised tumours at pH 5 in chemo-hyperthermia cancer therapy	[137]

**Table 6:** Sonodynamical triggers with biosensors to release API in drug carriers, with remarks listed.

Biosensor	API	Drug Carrier	Remarks	Ref.
TiO_2_	Ptx	TiO_2_/IONP/PEG-QDs	US duration control drug release profile	[138]
Porphyrin	Dox	PP	Enhanced local delivery and tumour suppression	[139]
NGR	Dox	DSPE/PEG_2k_/NGR	Triggered lipid bilayer to break down in antitumor effects	[140]
PFCMB	Dox	IONP/PEG/PFCMB	Deeper site targeting and precise delivery	[141]

**Table 7:** Sonodynamical triggers with biosensors with added redox and electro-ion stimuli in drug carriers, with remarks listed.

Trigger	Stimulus	Biosensor	API	Drug Carrier	Remarks	Ref.
IR780	Redox	TL	Ptx	DSPE-PEG_2k_-NH_2_/IR780/TL	US induced ROS for tumour growth inhibition and apoptosis	[142]
TiO_2_	Redox	PBE	Dox	TiO_2_-PBE	High local accumulation and tumour growth inhibition	[143]
TAPP	Electro-ion	CuS and Pt	H_2_O_2_	Pt-CuS/TAPP	Pt catalyses H_2_O_2_ for tumour hypoxia and cell apoptosis therapies	[144]

**Table 8:** Performance of the CuZn and Cu electrical biosensors in drug carriers with remarks.

Biosensor	API	Drug Carrier	Remarks	Ref.
CuZn	HAp	CuZn/G/Chi	Anti-bacterial activity increased, but osteoprogenitor cells were not cytotoxic	[150]
Cu MOF	miR-155	Au@Cu MOF	Current response change of 25 µA and the DPV detection limit of 0.35 fM	[151]
Cu MOF	Dox	(BTC)_2_/Cu_3_/IONP	Adsorbed 40.5% and released 85.5% at pH 5	[152]

**Table 9:** Electrical triggers with biosensors of API in drug carriers and remarks.

Biosensor	API	Drug Carrier	Remarks	Ref.
AT	Dex	Agr/Alg/AT	Enhanced cell viability and proliferation for neuroregenerative medicine	[154]
IONC	IDOi	IDOi/IONC	Synergistic effects on immuno-ablation cancer therapy with local magnetic field	[155]
PEGDMA	Vcm	Chi/IONP/PEGDMA	Controllable stimulation of DDS	[145]
Plt/PVA	RT	Plt/PVA/ PAA	Efficient transdermal DDS	[156]

**Table 10:** Electrical triggers with added enzymatic biosensors and their cleavage sites in drug carriers with test remarks.

Biosensor	Cleavage Site	Drug Carrier	Test Remarks	Ref.
MMP-2	Gly-Val	CS-Au-Pb/P-NH_2_/PANI ^1^	Electrochemical	[159]
MMP-2	Gly-Val	DNA-P/rDNA ^2^	Electrochemical	[160]
MMP-2	Gly-Val	PMUA/CB8/Ag/P2 ^3^	Electrochemical	[161]
MMP-2	Sulfhydryl P ^4^	NGQDs/sulfhydryl P/Ru@SiO_2_	Electrochemical	[157]
MMP-9	Leu-Met	MoS_2_/Si/Aβ_1–42_	Circulating protein by FET	[162]
MMP-9	Gly-Leu	Fe_3_O_4_/Ir(III)His-P	Fe_3_O_4_ by magnetic, Ir(III) by FRET	[163]
MMP-2/-9	Gly-Met	SiO_2_/rhodamine b/P-P ^5^	Rhodamine b by FRET	[165]
MMP-9	Gly-Met	Au/CSH/Gly-Met/ferrocene	DPV	[166]

Note: ^1^ P of P-NH_2_ is NH_2_-Gly-Gly-Lys-Gly-Arg-Val-Gly-Leu-Pro-Gly-Cys-SH, and its cleavage site seems to mostly be Gly-Val. ^2^ DNA-P is biotin-KKGRV-GLPGC-5′-CTA CTT ATG GCA GTG CTC GAA T-3′, and rDNA is 5′-ATT CG(Cy3)A GCA CT(BHQ2)G CCA-3′. ^3^ P of PMUA is FGPLGVRGKGGC-11 and P2 is FGGGASLWWSEKL. ^4^ Sulfhydryl P is labelled with a specific P sulfhydryl by the author. ^5^ P-P is PLGMWSRPLGMWSRPLGMWSR, and rhodamine b functions as a staining fluorescent dye and API.

## Data Availability

Not applicable.

## Abbreviations

AA, amino acids; AAm, acrylamide; Aβ, amyloid β_1-42_; AcDEX, acetalated dextran; ADAMTS5, a disintegrin and metalloproteinase with thrombospondin motifs 5; Agr, agarose; AI, ICAM-1 antibody; AIA, adjuvant-induced arthritis; Alg, alginate; AN, acrylonitrile; ANXA_1_, anti-inflammatory protein-annexin A_1_; API, active pharmaceutical ingredients; AT, aniline tetramer; ATP, adenosine 5′-triphosphate; Azo, azobenzene derivatives; BMP-2, bone morphogenetic protein-2; BSA, bovine serum albumin; C, cysteine; CB8, cucurbit[8]uril; Chi, chitosan; CS, carbon sphere; CSH, cysteamine hydrochloride; CTSK, cathepsin K; DDS, drug delivery system; Dex, dexamethasone; DMARD, disease-modifying anti-rheumatic drugs; DMOAD, disease-modifying osteoarthritis drugs; DMSA, meso-2,3-dimercaptosuccinic acid; DMSO, dimethyl sulfoxide; DNAA, DNA aptamers; DNA-P, DNA-peptide; Dox, doxorubicin; DPV, differential pulse voltammetry; DSPE, 1,2-distearoyl-sn-glycero-3-phosphoethanolamine; EA, electrostatic attraction; ECL, electro-chemo-luminescence; FET, field-effect transistor; FITC, fluorescein isothiocyanate; FRET, fluorescence resonance energy transfer; G, gelatine; GCTI, government clinical trial identifiers; Gly, glycine; GO, graphene oxide; GSH, glutathione; HA, hyaluronic acid; HE, haematoxylin and eosin; His, histidine; His-P, his-rich peptide; HSA, human serum albumin; IA, inflammatory arthritis; IDOi, indoleamine 2,3-dioxygenase inhibitors; I-κB, inhibitor of nuclear factor kappa B; IL, interleukin; IONC, iron-oxide-nanocube clusters; IONP, iron oxide NP; Ir(III) His-P, Ir[(2-phenylpyridine)_2_(His-P)] CF_3_SO_3_; K/BxN, T cell receptor transgene KRN and MHC class II molecule; Leu, leucine-enkephalin; Lys, lysine; Met, methionine-enkephalin; miR, microRNA; mHPMC, magnetic hydroxyl propyl methyl cellulose; MMP, matrix metalloproteinases; MMS, magnetic mesoporous silica; MNP, magnetic NP; MoS_2_, molybdenum disulphide; MPC, 2-methacryloyloxy-ethyl phosphorylcholine; MSC, mesenchymal stem cell; mTOR, mammalian target of rapamycin; nBMP-2, BMP-2 n-capsules; NF-κB, nuclear factor kappa-light-chain-enhancer of activated B cells; Mtx, methotrexate; NGQDs, nitrogen-doped graphene quantum dots; NGR, asparagine-glycine-arginine peptides; NIPAMAA, *N*-isopropylacrylamide-*co*-methacrylic acid; NIR, near infra-red; NLRP3, NOD-, LRR-, and pyrin domain-containing protein 3; NO, nitric oxide; NP, nanoparticles; NR, neutrophil-erythrocyte; NSAID, non-steroidal anti-inflammatory drugs; OA, osteoarthritis; P, peptides; PA, photoacoustic; PANI, polyaniline; PBAE, poly(β-amino ester); PBE, phenylboronic ester; PDA, polydopamine; PEG, polyethylene glycol; PEGDMA, PEG dimethacrylate; Peptide-P, peptide-pentynoic acid; PEtOx, poly(2-ethyl-2- oxazoline); PFCMB, perfluorocarbon gas microbubble; Phe, phenyl-alanine; PIPAMAA, poly[(*N*-isopropylacrylamide)-*co*-(methacrylic acid)]; Plt, pullulan; PMPC, MPC monomer and MMP cleavable peptide crosslinker; PMUA, peptide-mercaptoundecanoic acid; PN, polynucleotides; PP, porphyrin-phospholipid; PPS, poly(propylene sulphide); PRF, platelet-rich fibrin; PTT, partial thromboplastin time; Ptx, paclitaxel; PVA, polyvinyl alcohol; QAW, ANXA_1_ tripeptides; QCM-D, quartz crystal microbalance with dissipation; QDs, graphene-quantum dots; RA, rheumatoid arthritis; rDNA, report-DNA; redox, reduction-oxidation; RGD, arginine-glycine-aspartic acid; RMT, RGD-MMP-2/9-T; RMTQ, RGD-MMP-TAT-QAW; ROS, reactive oxygen species; RT, rivastigmine tartarate; Ru@SiO_2_, tris(bipyridine)ruthenium(II)-doped silica; *S. aureus*, Staphylococcus aureus; SD, Sprague-Dawley; SOD, superoxide dismutase; SPID, Sum of Pain Intensity Difference; SPIONs@Au; gold-coated superparamagnetic iron oxide nanoparticles; T-cell, thymus cell; TA, triamcinolone acetonide; TAT, transcription-transactivating protein; TATQ, TATQAW; Ted, tedizolid; TG-18, triglycerol monostearate; TIM, tumour immunosuppressive microenvironment; TME, tumour microenvironment; TNF, tumour necrosis factor; Tof, tofacitinib; TRP, transient receptor potential cation channel; UCNP, up-conversion luminescent NP; US, ultrasound; US-PA, ultrasound-guided photoacoustic; Val, valine; Vcm, vancomycin; WOMAC, and the Western Ontario and McMaster Universities Osteoarthritis Index.
